# The Role of HIV-1-Encoded microRNAs in Viral Replication

**DOI:** 10.3390/microorganisms12030425

**Published:** 2024-02-20

**Authors:** Ofira Carmi, Yosef Gotlieb, Yonat Shemer-Avni, Zvi Bentwich

**Affiliations:** 1 The Department of Medical Sciences, Faculty of Health Sciences, Ben-Gurion University of the Negev, Beer-Sheva 84105, Israel; 2 David Yellin College of Education, Jerusalem 91035, Israel; 3 The Shraga Segal Department of Microbiology Immunology and Genetics, Ben-Gurion University of the Negev, Beer-Sheva 84105, Israel; 4 Center for Tropical Diseases and AIDS, Ben-Gurion University of the Negev, Beer-Sheva 84105, Israel

**Keywords:** HIV-1, microRNA, deep sequencing, viral replication

## Abstract

microRNAs (miRNAs) are small non-coding RNAs (sncRNAs) that play an important role in the life cycle of human viruses. We sought to characterize human immunodeficiency virus 1 (HIV-1)-encoded miRNAs and determine their role in viral replication. Initially, a bioinformatic analysis was used to predict HIV-1-encoded miRNAs. Next, a representative number of these predicted sequences were verified using a miRNA microarray chip, reverse transcription PCR (RT-PCR), and the deep sequencing of RNA extracted from HIV-1-infected cells. Eight HIV-1-encoded sncRNA sequences conforming to the criteria that define miRNAs were identified in HIV-1-infected immortalized T cells and human primary CD4+ lymphocytes; five of the eight sequences have not been previously reported. Deep sequencing validated the presence of these virus-encoded miRNA sequences and uncovered large numbers of atypical sncRNA sequences, lacking characteristics of conventional miRNAs. We named these sequences small RNAs (smRNAs). The overexpression of four candidate HIV-1-encoded miRNAs and silencing of two smRNAs significantly increased HIV-1 viral replication. Our study uncovered novel HIV-1-encoded sncRNAs that, upon deregulated expression, alter viral titers in HIV-1-infected cells, suggesting that miRNAs and smRNAs play an important role in regulating viral replication. Future studies may reveal the function of HIV-1-encoded sncRNAs and their possible implications for diagnosis and treatment.

## 1. Introduction

While substantial progress has been made in the drug treatment of human immunodeficiency virus 1 (HIV-1) in recent decades, present therapies are unable to eliminate the virus from the infected host. Mechanisms responsible for maintaining the virus in a latent state and regulating its replication remain unclear. However, it has been suggested that both HIV-1 and host-encoded microRNAs (miRNAs) play central roles in the regulation of these processes, although their modes of action have not yet been completely elucidated [1,2,3,4,5,6,7].

miRNAs are small non-coding RNA (sncRNA) molecules that play a crucial role in the regulation of gene expression via post-transcriptional suppression and translation inhibition, resulting in the downregulation of gene expression and, accordingly, affect the modulation of numerous biological functions [8,9]. Various studies indicate that miRNAs are situated in the intronic and exonic regions of coding and non-coding genes, as well as in intragenic regions. 

Hundreds of eukaryotic cellular miRNAs found to function as post-transcriptional regulators of viral genes have been identified [8,10]. Some of these host cell miRNAs play a role in interfering with the establishment of latent HIV-1 infection [11,12], while others promote cell activation and HIV-1 latency [13,14]. Interestingly, HIV-1-infected cells differentially express cellular miRNAs depending on whether the virus is productive or latent [15], thereby marking them as possible candidates for biomarkers of disease progression [16,17]. In contrast to the numerous studies identifying host cell miRNAs and elucidating their role in the regulation of HIV-1 infection, there remains a paucity of information regarding HIV-1-encoded miRNAs and their role in controlling viral replication [1,18,19,20].

In addition to well-defined miRNAs, there is emerging evidence for the existence of noncanonical sncRNAs, derived from longer non-coding RNAs. These noncanonical sncRNAs range in size from 15 to 50 nts—whereas miRNAs are 18–22 nts in length [21]—and might not necessarily contain an RNA hairpin motif, thus deviating from the standard definition of a miRNA.

The objectives of this study were to identify and characterize sncRNAs encoded by HIV-1 and clarify their potential role in the viral life cycle. The characterization of sncRNAs in HIV-1 and their effects on host cells at different stages of viral infection may greatly contribute to an improved understanding of the HIV-1 life cycle and the pathogenesis of acquired immunodeficiency syndrome (AIDS), thus leading to the development of novel approaches for the therapy of HIV-1 infection. 

Our research strategy entailed the following: (1) confirming the potential role of virus-encoded sncRNAs in controlling viral replication, (2) identifying both previously described and novel miRNA-like structures in the HIV-1 genome via bioinformatic predictions, (3) performing reverse transcription PCR (RT-PCR) to verify the presence of predicted miRNA transcripts in infected cells and to ascertain whether these structures display characteristics associated with canonical miRNAs, (4) implementing a deep sequencing of HIV-1-infected cells to identify all HIV-1-encoded miRNAs, in addition to searching for noncanonical small RNAs (smRNAs) and determining their levels of expression, and (5) testing the effects of candidate miRNAs and smRNAs on HIV-1 viral replication.

## 2. Materials and Methods

### 2.1. Cells

Monolayer adherent HEK293T (ATCC CRL-3216) [22] and HeLa-CD4-LTR-β-gal cells, expressing the β-galactosidase gene under the regulation of the transactivation response element (TAR) [23], were grown in DMEM. The T-lymphocyte cell line, T1 (CEMx174) (ATCC CRL-1991) [24], was grown in RPMI 1640 medium. All media were supplemented with 10% (*v*/*v*) fetal calf serum, 0.3 g/Liter of L-glutamine, and 100 units/mL of penicillin/streptomycin (Biological Industries, Beit Haemek, Israel). 

### 2.2. Bioinformatic Prediction of miRNAs

Rosetta Genomics developed bioinformatic prediction tools to detect miRNAs in the human genome that might have been overlooked by past methods, which detected only conserved hairpin structures [25]. Their approach comprised the following steps: (i) computationally scanning the entire human genome for hairpin structures, (ii) annotating all hairpins for conserved, repetitive, and protein-coding regions, (iii) scoring hairpins by thermodynamic stability and structural features, (iv) determining the expression of computationally predicted miRNAs using a high-throughput miRNA microarray in various human tissues, and (v) validating the sequence of predicted miRNAs that yielded strong signals on the microarray using a new sequence-directed cloning and sequencing method. For our study, the tool was used to scan the HIV-1 genome for hairpin structures. All miRNA sequences that were homologous between the viral and the human genome were removed from the analysis.

### 2.3. Preparation of HIV-1 Virus Stocks

VSV-G pseudotyped viruses were produced through a co-transfection of HEK293T cells with the env-defective HIV-1 pSVC2.1 expression vector and the pCMV-VSV-G envelope vector (Addgene, Watertown, MA, USA, plasmid #8454). Culture supernatants were harvested 48 h post transfection, titrated on HeLa-CD4-LTR-β-gal cells, and stored at −80 °C. VSV-G-pseudotyped HIV-1 replication-defective viral particles were produced and employed according to standard operating procedures for working with lentiviral vectors, which included carrying out experiments in a BSL2 laboratory containing a Class II Biosafety Cabinet.

### 2.4. The pLKO.1 Cloning Vector Lentivirus System 

Oligos of target sequences to be knocked down or overexpressed by short hairpin RNAs (shRNA) (Table S1) were annealed and inserted into the pLKO.1 puro vector (Addgene, plasmid #8453) according to the protocol published by Addgene (https://www.addgene.org/protocols/plko/#C, accessed on 20 January 2023) [26]. Viral particles were produced through a co-transfection of HEK293T cells with the shRNA expression vector, the lentiviral packaging vector, pCMV-dR8.2 dvpr (Addgene, plasmid #8455), and the pCMV-VSV-G envelope plasmid (Addgene, plasmid #8454). Lentivirus-containing supernatants were collected 48 h post transfection. 

### 2.5. Tough Decoy (TuD) Lentivirus System 

The TuD RNA expression was used to validate predicted candidate structures. A series of oligonucleotide pairs (Table S2) were annealed and cloned into the pmU6-TuD-shuttle plasmid digested with BsmBI [27]. Each mU6-TuD RNA cassette, flanked by BamHI–EcoRI sites, was subcloned into the BamHI/EcoRI-digested pLSP lentiviral vector (kindly provided by Prof. Yinon Ben-Neriah, Faculty of Medicine, The Hebrew University of Jerusalem, Israel). The resulting construct was co-transfected with pCMV-dR8.2 dvpr and pCMV-VSV-G into HEK293T cells. Lentiviral supernatants were collected 48 h post transfection [27].

### 2.6. Microarrays

Complementary RNA synthetic transcript (cRNA) libraries from both infected and uninfected control cells were prepared and labeled with Cyanine 5-CTP (Cy5-CTP) or Cyanine 3-CTP (Cy3-CTP) using the Low-Input Linear Amplification Kit (Agilent Technologies, Santa Clara, CA, USA) [28]. One microgram of labeled cRNA was loaded onto an Agilent Technologies MIRCHIP miRNA array adapted for 45 mer oligonucleotides that matched the HIV-1 miRNA predictions. The labeled cRNA from the infected and control samples were processed using Agilent Technologies’ in situ Hybridization Reagent Kit. The microarray was scanned using the Agilent LP2 laser scanner at a 0.10 μm resolution.

### 2.7. RT-PCR

Total RNA was isolated from cultured T1 cells using the EZ-RNA II Total RNA Isolation Kit (Biological Industries, Beit Haemek, Israel). The total RNA extracted from CD4+ T lymphocytes isolated from three donors and infected ex vivo with the DH12 strain of HIV-1 [29] was kindly provided by Prof. Tomozumi Imamichi (NIH, Laboratory of Human Retrovirology and Immunoinformatics, Frederick, MD, USA).

cDNA was synthesized using the miScript II RT Kit (QIAGEN, Germantown, MD, USA). The QuantiTect SYBR Green RT-PCR Kit (QIAGEN, Germantown, MD, USA) was used according to the manufacturer’s instructions. The quantification was normalized to the housekeeping genes U6 or β-actin. Primers are listed in Table S3. The RT-PCR products were run on a 2% agarose gel to detect their size. 

### 2.8. Deep Sequencing

Total RNA from infected and uninfected control T1 cells was isolated as previously described for RT-PCR. A cDNA library was generated; resulting fragments were run on an agarose gel, and fragments smaller than 36 bp were extracted from the gel and loaded onto an Illumina Genome Analyzer (Illumina Inc., San Diego, CA, USA). A bioinformatic analysis was conducted to identify HIV-1 genome sequences by aligning the cDNA library sequences to the genomic sequence of the HXB2 strain using the miRNAkey sofware [30]. Sequences found to be homologous between human and HIV-1 miRNAs were removed.

### 2.9. Transduction with Lentiviruses

The T1 cells (2 × 10^6^) were transduced overnight with 1 mL of lentiviral supernatants in a 24-well tissue culture plate. Antibiotic selection was performed 72 h post infection with 1 ug/mL puromycin (Sigma-Aldrich, St. Louis, MO, USA). 

## 3. Results

### 3.1. The Effect of Dicer Knockdown (KD) on HIV-1 Viral Replication

**Figure 1:**
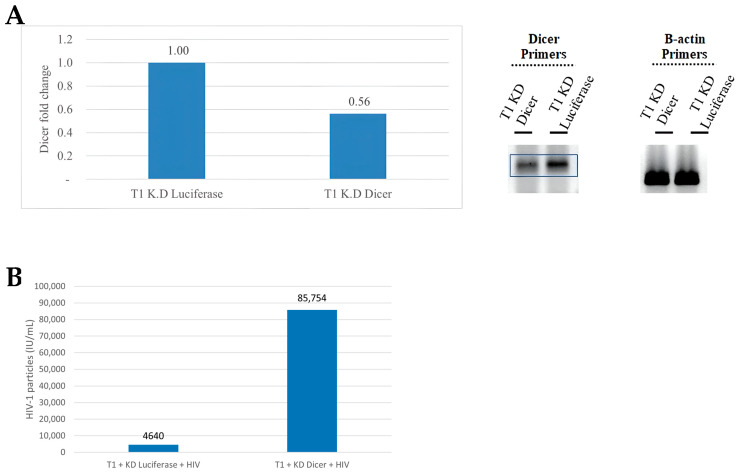
The effect of Dicer knockdown (KD) on HIV-1 replication. (**A**) shRNA targeted to Dicer resulted in a decreased Dicer expression. RT-PCR products were normalized to the β-actin housekeeping gene (**left panel**). PCR products were analyzed on an agarose gel (**right panel**). (**B**) The number of HIV-1 particles was measured in T1 cells transduced with control sh-Luciferase (K.D Luciferase) or shDicer (K.D Dicer). The number of cells was normalized between groups. Representative experiment, *n* = 2.

To demonstrate the possible role of putative HIV-1-derived miRNAs on viral replication in infected cells, we initiated our study by confirming previous work showing that the KD of Dicer results in increased HIV-1 viral replication in infected cells [31,32]. Past reports have demonstrated that knocking down Dicer decreases the production of HIV-1-encoded miRNAs [6]. We transduced T1 cells with a previously validated shRNA targeted to Dicer (K.D Dicer) [33] while control cells were transduced with a corresponding shRNA viral vector targeted to the luciferase gene (K.D Luciferase). RT-PCR revealed a 50% reduction in Dicer expression in KD T1 cells as compared to that of the control cells (Figure 1A, left panel). In agreement with the RT-PCR analysis, RT-PCR products run on an agarose gel displayed weaker band signals in Dicer KD cells than in control K.D Luciferase cells (Figure 1A, right panel). The Dicer KD and control cells were then infected with HIV-1 viral particles. Two days post the HIV-1 infection, the number of viral particles in the supernatant was determined via titration on HeLa-CD4-LTR-β-gal cells. Dicer KD HIV-1-infected cells produced 85,754 IU as compared to 4460 IU in control cells, which is a 19.2-fold increase in the number of viral particles (Figure 1B). Since Dicer is required for canonical miRNA biogenesis [34], our results demonstrate that a marked reduction in miRNA processing, through the silencing of Dicer, leads to a substantial increase in viral replication. This confirms previous studies and strongly suggests an important role for miRNAs in controlling viral replication.

### 3.2. Validation of Bioinformatically-Predicted miRNA Sequences in the HIV-1 Genome

**Figure 2:**
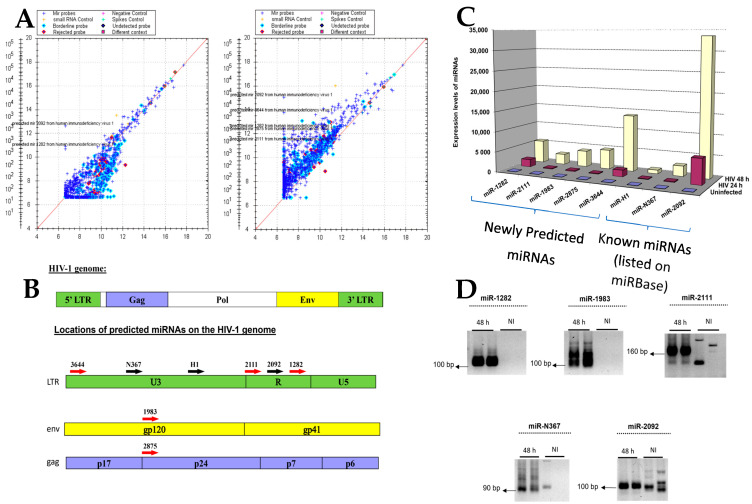
Validation of bioinformatically-predicted microRNAs (miRNAs) in the HIV-1 genome. (**A**) Microarray expression profiling of bioinformatically-predicted HIV-1-encoded miRNAs in RNA obtained from T1 cells 24 h (**left panel**) and 48 h (**right panel**) post HIV-1 infection. (**B**) **Top panel**: a schematic representation of the HIV-1 genome. **Bottom panel**: the positions of eight predicted HIV-1-encoded miRNAs in the viral genome (five new predictions are indicated by red arrows; the three miRNAs listed on the miRbase website are indicated by black arrows). (**C**) Expression levels of predicted HIV-1-encoded miRNAs 24 and 48 h post HIV-1 infection. (**D**) RT-PCR products of three newly predicted miRNA sequences (1282, 1983, 3644) and two known miRNAs (N367 and 2092, also known as miR-TAR-3p) displayed on a 2% agarose gel. NI, non-infected.

cDNA synthesized from RNA extracted from T1 cells, one and two days post HIV-1 infection, was loaded onto Rosetta Genomics diagnostic chips containing nucleotide sequences designed to hybridize with predicted miRNAs encoded by the HIV-1 genome (Figure 2A). Eight candidate miRNAs predicted via bioinformatic analysis were identified on the chip (Figure 2B and Table S4) and were, therefore, considered to be prospective HIV-1-encoded miRNAs. Three of these miRNAs were previously described in the literature [10], although they, along with the newly discovered sequences, had not yet been profiled. Next, we measured the expression levels of all eight candidate miRNAs via RT-PCR, 24 and 48 h post HIV-1 infection. The expressions of the two newly discovered miRNAs (miR-1282 and miR-3644) and one previously reported miRNA (miR-2092) were upregulated 24 h post infection as compared to uninfected cells, and notably, all eight miRNAs were upregulated 48 h after infection (Figure 2C). Next, RT-PCR was performed to validate the microarray and miRNA expression results. Primers ranging from 20 to 28 nts were used to identify nucleotide sequences corresponding to the length of conventionally defined miRNAs (Table S3). RT-PCR products were expected to be approximately 100 bps, corresponding to the length of the miRNA together with 60–80 base pairs added via oligo dT priming. RT-PCR confirmed the chip data for all eight candidates. Figure 2D illustrates the validation of three newly discovered miRNAs (miR-1282, miR-1983, and miR-2111) and two previously predicted sequences (miR-N367 and miR-2092). Surprisingly, the RT-PCR on miR-2111 revealed a 160 bp PCR product instead of the expected 100 bps, which could be interpreted as an unprocessed miRNA. Nevertheless, given the presence of this sequence only in infected cells, its location adjacent to the previously described miR-2092, and its upregulation 48 h post HIV-1 infection, it was retained for further study. 

Taken together, miR-1282, miR-1983, and miR-2111—detected only in infected cells via RT-PCR and with upregulated expressions following HIV-1-infection—were considered likely candidates, whereas miR-2875, miR-H1, and miR-3644, observed in both infected and uninfected cells, were considered less probable HIV-1-encoded miRNA candidates.

### 3.3. Identifying the Locations of miRNAs via Deep Sequencing 

We then performed a deep sequencing of the HIV-1-infected T1 cells to further assess candidate sequences corresponding to the criteria defining miRNAs. Total RNA was extracted from HIV-1-infected cells four and eight days post infection and from uninfected controls. 

The deep sequencing resulted in numerous sequences that were identified as potential HIV-1-derived miRNAs. These sequences were aligned to the genomic sequence of the HXB2 strain of HIV-1. They were also matched to sequences predicted in our bioinformatic analysis (confirmed via microarray) and to those listed in the miRNA database, miRBase. 

**Figure 3:**
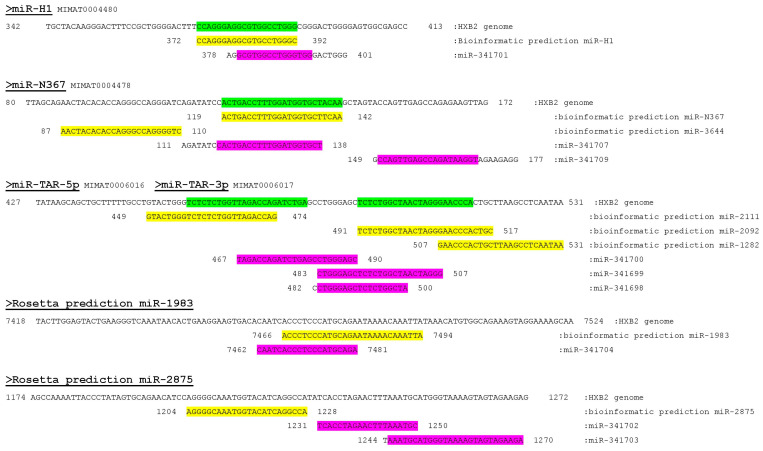
Locations of miRNAs in the HIV-1 genome identified via deep sequencing relative to those predicted by Rosetta Genomics. The sequences in green are miRNAs listed in miRbase. Those in yellow were bioinformatically predicted using Rosetta Genomics’ microarray. Sequences highlighted in pink were discovered via deep sequencing and were studied further.

Figure 3 displays the locations of segments identified via deep sequencing (highlighted in pink) in relation to their positions in the viral genome and miRbase-indexed miRNAs (highlighted in green) as well as their locations with reference to the eight predicted miRNAs detected on the microarray (highlighted in yellow). Of interest, miR-H1 (indexed in miRBase) is situated near or overlaps with four sequences identified via deep sequencing, of which one, miR-341701, was selected for further study. The miRBase-listed miR-N367 is situated near nine sequences identified via deep sequencing. Three of these sequences were also identified via RT-PCR; two were selected for further study (miR-341707 and miR-341709). It should be noted that miR-341707 was detected twice with a sequence that differed by one nucleotide. The location of the miRNA known in miRBase as miR-TAR-3p/5p is in the vicinity of ten sequences identified via deep sequencing; three were chosen for further study (miR-341698, miR-341699, and miR-341700). The microarray-predicted sequence, miR-1983, overlapped with one sequence detected via deep sequencing, namely, miR-341704, which was chosen for further study. The microarray study predicted miR-2875, which was located proximate to two sequences discovered via deep sequencing, which were chosen for further study (miR-341702 and miR-341703). Altogether, nine miRNAs (listed in Table S5)—which conformed to the conventional definition of a miRNA—were further investigated based on the convergence between sequences indexed in miRBase, the microarray data, and the deep sequencing results. 

### 3.4. Confirmation of miRNA Expression in HIV-1-Infected Cells

**Figure 4:**
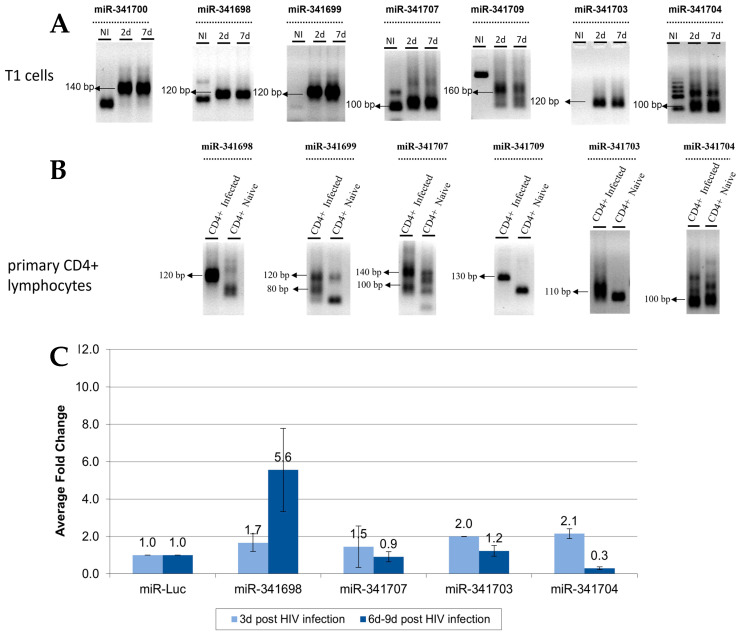
Confirmation of miRNA expressions in an HIV-1-infected T-lymphocyte cell line and in primary human T cells. Total RNA was extracted from HIV-1-infected (**A**) immortalized T cells (T1) or (**B**) primary CD4+ lymphocytes. RT-PCR was performed using primers with the seven miRNA sequences highlighted in pink in Figure 2. (**C**) HIV-1 viral production in cells following miRNA overexpression. T1 cells were transduced with lentiviral vectors harboring four candidate miRNAs and were then infected with HIV-1. Three days and six to nine days post HIV-1 infection, culture supernatants were collected, and the number of viral particles was quantified and compared to that of the control cells overexpressing shLuc.

To confirm the expressions of the nine putative miRNA sequences discovered through deep sequencing, we performed RT-PCR on RNA extracted from HIV-1-infected T1 cells and primary CD4+ T lymphocytes using the primers listed in Table S3. The sizes of the expected products were calculated by combining the length of the primer (20–28 nts) with the 60–80 nts added through the cDNA library preparation. Thus, the resulting products were expected to be approximately 100 nts in length. RT-PCR detected seven of nine miRNAs in T1 cultured cells, whereas the RT-PCR products of RNA isolated from uninfected cells were either absent or were of unexpected size (Figure 4A). In HIV-1-infected primary CD4+ lymphocytes, six of nine RT-PCR products were of expected length (Figure 4B). Based on the size of the RT-PCR products in both cultured and primary cells, the strong signals they displayed, and their expressions in infected cells only, we continued investigating four of the predicted miRNA sequences that were not previously identified, namely, miR-341698, miR-341707, miR-341703, and miR-341704. 

### 3.5. The Role of Candidate miRNAs in Viral Infection 

To determine the roles of the four aforementioned miRNAs in viral replication, we overexpressed these miRNAs in T1 cells using a lentiviral construct, infected the cells with HIV-1 viral particles, and then monitored the number of viral particles produced over time. Notably, three days post HIV-1 infection, the overexpression of miR-341703 and miR-341704 led to a two-fold increase in the number of viral particles, compared to the control cells. The overexpression of miR-341698 and miR-341707 resulted in more modest 1.7-fold and 1.5-fold increases in viral replication, respectively. At 6–9 days post infection, the overexpression of miR-341698 increased viral replication 5.6-fold, whereas upon the overexpression of the remaining three miRNAs, viral particle production returned to that of the control levels or less (Figure 4C). These results demonstrate that upon the overexpression of previously described miRNAs, as well as newly discovered miRNAs, there was a trend toward increased HIV-1 replication in the first few days post infection. However, one week following HIV-1 infection, viral replication was reduced to control or subcontrol levels, possibly via suppression by cellular mechanisms.

### 3.6. Characterization of smRNA Sequences in HIV-1-Infected Cells

We repeated the deep sequencing experiment to identify additional miRNAs in HIV-1-infected cells. In this second deep sequencing run, the bioinformatic algorithm detected a wider range of sncRNAs than in the first sequencing assay, which identified only typical miRNAs of approximately 28 base pairs presented in a stem-loop structure. Although there were a sizeable number of sncRNA reads (75,048), typical miRNA structures were not detected. We referred to these non-canonical sncRNAs as smRNAs. 

**Figure 5:**
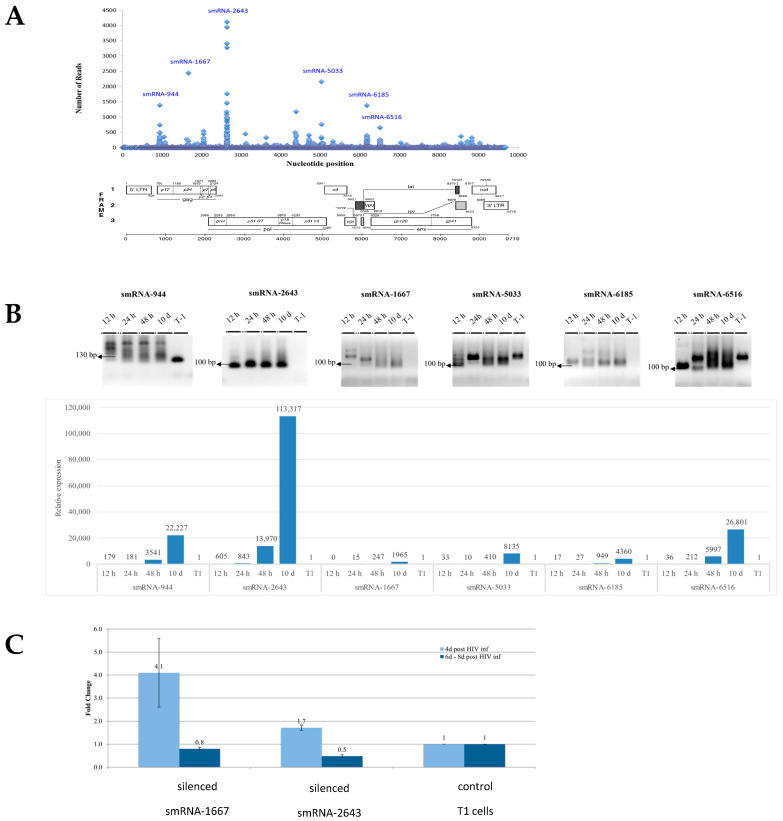
Identification and validation of HIV-1-encoded small RNAs (smRNAs). (**A**) Deep sequencing of RNA extracted from HIV-1-infected T1 cells identified smRNAs that differed in length and structure from conventional miRNAs; the numbers of reads of smRNAs and their locations on the HIV-1 genome. (**B**) Time kinetics of sm-RNA expression post HIV-1 infection. Upper panel: RT-PCR products run on an agarose gel. Lower panel: relative expression as compared to that of uninfected T1 cells, measured via RT-PCR. Representative experiment, *n* = 3. (**C**) Two HIV-1-encoded smRNAs were knocked down in T1 cells. The cells were subsequently infected with HIV-1 at an MOI of 0.5. The number of viral particles produced was measured four and eight days post infection. *n* = 2, mean ± SD.

In the upper panel in Figure 5A, the vertical axis displays the number of reads that were obtained for each sequence with each dot on the graph representing a 20–36 nt sequence; the horizontal axis represents the nt position along the HIV-1 genome. The lower panel in Figure 5A displays the viral genes and their positions on the HIV-1 genome. Interestingly, smRNA sequences that contained numerous reads were concentrated in very specific regions of the HIV-1 genome. We focused our efforts on studying the six smRNA sequences with the highest number of reads (Table 1). 

Using RT-PCR, we measured the time kinetics of the expressions of these six smRNAs 12 h to 10 days post HIV-1-infection. miR-181a, a cellular housekeeping gene, was used as a positive control and was observed in both naïve and infected cells. Using primers to the candidate smRNAs (Table S3), a pattern of RT-PCR products was revealed in HIV-1-infected cells that differed substantially from the uninfected T1 cells (Figure 5B, upper panel) and, importantly, their expression increased with time, as measured via RT-PCR (Figure 5B, lower panel).

We then further characterized the smRNAs that (a) had been bioinformatically predicted, (b) revealed clear RT-PCR products of expected sizes, (c) resulted in the largest number of reads in the deep sequencing, and (d) were expressed only in infected cells. Two smRNAs met all of these criteria: smRNA-1667 with 2448 reads and smRNA-2643 with 4122 reads. 

### 3.7. Effect of HIV-1-Associated smRNAs on the Suppression of Viral Replication

To further characterize the role of these smRNAs, we employed TuD RNAs, a system used to silence specific miRNAs. The TuD molecule is a stabilized stem-loop containing two miRNA binding domains that target miRNA sequences and prevent the miRNAs from binding to mRNA and silencing gene expression. 

In this set of experiments, T1 cells were transduced with a lentivirus containing TuD molecules targeting smRNA-1667 and smRNA-2643. Two weeks after puromycin selection, the cells were infected with HIV-1 at an MOI of 0.5. The cell supernatant was collected four and six to eight days post infection, and the viral replication levels were ascertained (Figure 5C). The most conspicuous change in viral replication occurred four days post HIV-1 infection upon the inhibition of smRNA-1667. A 4.1-fold increase in viral replication was observed as compared to that of the control HIV-1-infected T1 cells in the absence of a TuD molecule. The inhibition of smRNA-2643 resulted in a minor increase of 1.7 folds upon HIV-1 infection. One week post infection, the number of viral particles either returned to control or decreased levels, possibly indicating a delayed, yet perhaps expected, cellular suppression of increased viral titer. These results indicate that, in contrast to the miRNAs discovered in this study of which their overexpression increase viral production, smRNAs tend to suppress HIV-1 replication.

## 4. Discussion

In this study, we attempted to identify and characterize HIV-1-derived miRNAs by asking the following questions: Is there an HIV-1-encoded miRNA expression in virus-infected cells? If so, do these molecules play a role in controlling viral replication?

miRNA involvement in HIV-1 viral production was first suggested from experiments in our study in which the silencing of Dicer in HIV-1-infected cells, using a knockdown construct verified in previous publications, resulted in an 18-fold increase in viral replication, as measured by the number of infectious particles. The significance of this finding is that Dicer—the protein responsible for the central enzymatic activity in the formation of essentially all miRNAs—plays a predominately inhibitory role in viral replication. To rule out off-target effects, it would be prudent to repeat the experiments performed herein using additional Dicer knockdown constructs. 

miRNA microarray chip analysis, validated via RT-PCR, identified eight candidate sequences in HIV-1-infected cells that were absent in uninfected cells. Of these eight sequences, three have been previously described [35], and five miRNAs were newly discovered in our study. We aimed to observe the presence of candidate miRNAs uncovered by the microarray data and, indeed, were able to detect these miRNAs using RT-PCR. 

Following two rounds of deep sequencing on HIV-1-infected cells, thousands of short RNA sequences, limited to specific areas of the viral genome, were identified. Some of these sequences conformed to typical miRNAs in length and structure, whereas others exceeded 30 nts in length and did not contain a hairpin loop, thereby diverging from the conventional definition of a miRNA. The presence of miRNAs identified in the first round of deep sequencing was further confirmed via RT-PCR in a cultured T cell line and, more importantly, in primary CD4+ T lymphocytes that were infected ex vivo. Further validation might be performed by demonstrating the presence of our candidate miRNAs in lymphocytes isolated from HIV-1-infected patients or by infecting T1 cells with clinical, wild-type strains of HIV-1 isolated from patients. 

Indications of the miRNA regulation of HIV-1 replication, coupled with numerous miRNAs associated with HIV-1 infection, prompted us to probe their mechanism of action. Interestingly, upon overexpressing the identified miRNA sequences in HIV-1-infected cells, a significant increase in the number of viral particles was observed, further reinforcing the premise that miRNAs play a supporting role in viral replication. 

The second round of deep sequencing produced large numbers of smRNAs in which sequences with the highest numbers of reads were centered in specific regions of the HIV-1 genome. When activity of these sequences was neutralized in HIV-1-infected cells using the TuD system, viral production was increased. These results might be corroborated by observing whether a decreased viral production is observed upon the overexpression of smRNAs. Our experiments clearly indicate that these newly characterized smRNA structures play a role in the biological processes of viral production albeit opposite to the function of the novel miRNAs investigated herein. 

To elucidate the effects of the newly discovered miRNAs and smRNAs on host cell gene expression, it would be most interesting to perform gene expression profiling on HIV-1-infected cells following the overexpression and knockdown of our candidate sncRNAs. Host cell gene expression profiling was out of the scope of the present study; however, the identification of genes that are differentially expressed in HIV-1-infected, sncRNA-engineered cells would shed light on their mechanisms of action, especially in regard to the unconventional smRNAs. 

Our findings join a series of previous works elucidating the role of miRNAs in the HIV-1 life cycle [1,5,36,37,38]. The first miRNA described in the HIV-1 genome was a stem-loop structure found in the viral gene, Nef (termed miR-N367) [5]. This study claimed that miR-N367 targets the HIV-1 LTR promotor, and consequently, it was proposed that its function is to reduce viral expression following infection. Two additional groups repeated this study yet were unable to corroborate this claim [39,40]. Subsequently, two viral siRNAs located in the Rev Response Element (RRE) were identified [36], which, when released from the RRE, neutralize mRNA and inhibit the expression of the Rev gene. However, a subsequent study maintained that the hairpin structures of these two siRNAs contained 197 base pairs and, therefore, were too large to correspond to functional siRNAs [41]. 

The TAR element—an additional viral gene—was studied as a prospective location of miRNAs [3,4,6]. This sequence is found in the 5’ LTR and has a central role in regulating the expressions of viral proteins. More recently, it has been proposed that the TAR DNA-binding protein inhibits the expressions of the cellular ERCC1 and IER3 proteins, which play important roles in apoptosis. Therefore, the suppression of genes encoding these proteins provides a significant advantage for viral production in infected cells [4]. In our study, the TAR-associated miRNAs were detected only at low frequency in one of the two deep sequencing experiments.

We found that, following a global disruption of miRNA processing via Dicer silencing, viral production is increased, suggesting that the net effect of miRNAs on HIV-1 is the inhibition of viral production. This effect is in agreement with the observed increase in viral replication upon smRNA silencing. This finding does not necessarily contradict our data in which the overexpression of specific miRNAs stimulated viral particle production, since each HIV-1-encoded sncRNA might affect viral (and host) genes in a unique fashion. Our results suggest a heterogeneous response of HIV-1 replication to the expression of individual HIV-1-encoded sncRNAs.

Our study lends support for the possibility that miRNAs and smRNAs, which share many characteristics with miRNAs, are involved in HIV-1 infection. Further studies must be carried out to definitively establish the roles of HIV-1-derived miRNAs and the intriguing smRNAs on viral production and host cell gene expression. Future studies along these lines could provide directions for new diagnostic tools and therapeutic modalities for the treatment of AIDS.

## Figures and Tables

**Table 1:** smRNAs revealed via second deep sequencing run.

Name	Sequence	Number of Reads	No. of bps
smRNA-944	AAACATCAGAAGGCTGTAGACAAATACTGGGA	1393	32
smRNA-2643	AAAGCATTAGTAGAAATTTGTACAGAGATGGA	4122	32
smRNA-1667	TTAGAGACTATGTAGACCGGT	2448	21
smRNA-5033	TAGGGATTATGGAAAACAGATGGC	2164	24
smRNA-6185	TTAATTGATAGACTAATAGAAAGAGCAGA	1381	29
smRNA-6516	AATGACATGGTAGAACAGATGCATGAGGAT	657	30

## Data Availability

Data were obtained from a third party and are available from the authors with the permission of the third party.

## Supplementary Materials

The following supporting information can be downloaded at https://www.mdpi.com/article/10.3390/microorganisms12030425/s1: Table S1: Table of the oligonucleotides of target sequences for the knockdown or overexpression of shRNAs; Table S2: Table of oligonucleotides targeted for the TuD knockdown of small RNAs (smRNAs); Table S3: Table of RT-PCR primers; Table S4: Rosetta Genetics sample numbers and predicted sequences; Table S5: miRNA sequences predicted in the first deep sequencing run.
