# Supplementary material for: The Role of HIV-1-Encoded microRNAs in Viral Replication
Source: Microorganisms. 2024 Feb 20;12(3):425. doi: 10.3390/microorganisms12030425 (PMC10971904; doi:10.3390/microorganisms12030425)
Supplement: Supplementary file 1 [file microorganisms-12-00425-s001.zip › microorganisms-2831947-supplementary.pdf]

Table S1

Oligonucleotides of target sequences for knockdown or overexpression of shRNAs

	Sequence
miR-341707 F (sh-N367)	CCGGTCACTGACCTTTGGATGGTGCTCTCGAGAGCACCATCCAAAGGTCAGTGTTTTTG
miR-341707 R (sh-N367)	AATTCAAAAACACTGACCTTTGGATGGTGCTCTCGAGAGCACCATCCAAAGGTCAGTGA
miR-341704 F (sh-1983)	CCGGTCAATCACCCTCCCATGCAGACTCGAGTCTGCATGGGAGGGTGATTGTTTTTG
miR-341704 R (sh-1983)	AATTCAAAAACAATCACCCTCCCATGCAGACTCGAGTCTGCATGGGAGGGTGATTGA
miR-341703 F (sh-2875)	CCGGTAAATGCATGGGTAAAAGTAGTAGAAGACTCGAGTCTTCTACTACTTTTACCCATGCATTTTTTTTG
miR-341703 R (sh-2875)	AATTCAAAAAAAATGCATGGGTAAAAGTAGTAGAAGACTCGAGTCTTCTACTACTTTTACCCATGCATTTA
miR-341698 F (sh-TAR)	CCGGTCTGGGAGCTCTCTGGCTACTCGAGTAGCCAGAGAGCTCCCAGTTTTTG
miR-341698 R (sh-TAR)	AATTCAAAAACTGGGAGCTCTCTGGCTACTCGAGTAGCCAGAGAGCTCCCAGA
sh-Dicer (designed for pLKO.1 system)	Dharmacon™, CAT# RHS3979-200804805
sh-Luciferase	TTACGCTGAGTACTTCGA

Table S2

Oligonucleotides targeted for TuD knockdown of small RNAs (sm-RNAs)

miRNA binding site	Sequence
smRNA-2643-F	CATCAACTCCATCTCTGTACAAATTTCTAATCTCTAATGCTTTCAAGTATTCTGGTCACAGAATACAACTCCATCTCTGTACAAATTTCTAATCTCTAATGCTTTCAAG
smRNA-2643-R	TCATCTTGAAAGCATTAGAGATTAGAAATTTGTACAGAGATGGAGTTGTATTCTGTGACCAGAATACTTGAAAGCATTAGAGATTAGAAATTTGTACAGAGATGGAGTT
smRNA-1667-F	CATCAACACCGGTCTACAATCTTAGTCTCTAACAAGTATTCTGGTCACAGAATACAACACCGGTCTACAATCTTAGTCTCTAACAAG
smRNA-1667-R	TCATCTTGTTAGAGACTAAGATTGTAGACCGGTGTTGTATTCTGTGACCAGAATACTTGTTAGAGACTAAGATTGTAGACCGGTGTT

Table S3

RT-PCR primers

	**Primer**	**Sequence**
Bioinformatics/ Microarray	miR-H1	CCAGGGAGGCGTGCCTGGGC
	miR-N367	ACTGACCTTTGGATGGTGCTTCAA
	miR-1282	GAACCCACTGCTTAAGCCTCAATAA
	miR-1983	ACCCTCCCATGCAGAATAAAACAAATTA
	miR-2092	TCTCTGGCTAACTAGGGAACCCACTGC
	miR-2111	GTACTGGGTCTCTCTGGTTAGACCAG
	miR-2875	AGGGGCAAATGGTACATCAGGCCA
	miR-3644	AACTACACACCAGGGCCAGGGGTC
Deep sequencing I	miR-341698	CTGGGAGCTCTCTGGCTA
	miR-341699	CTGGGAGCTCTCTGGCTAACTAGGG
	mir-341700	TAGACCAGATCTGAGCCTGGGAGC
	miR-341701	GCGTGGCCTGGGTGG
	mir-341702	TCACCTAGAACTTTAAATGC
	miR-341703	AAATGCATGGGTAAAAGTAGTAGAAGA
	miR-341704	CAATCACCCTCCCATGCAGA
	miR-341707	CACTGACCTTTGGATGGTGCT
	miR-341709	CCAGTTGAGCCAGATAAGGT
Deep sequencing II	smRNA-944	AAACATCAGAAGGCTGTAGACAAATACTGGGA
	smRNA-1667	TTAGAGACTATGTAGACCGGT
	smRNA-2643	AAAGCATTAGTAGAAATTTGTACAGAGATGGA
	smRNA-5033	TAGGGATTATGGAAAACAGATGGC
	smRNA-6185	TTAATTGATAGACTAATAGAAAGAGCAGA
	smRNA-6516	AAAATGACATGGTAGAACAGATGCATGAGG
	hsa-miR-181a	AACATTCAACGCTGTCGGTGAGT
	pLKO.1 sequencing prime	CAAGGCTGTTAGAGAGATAATTGGA

Table S4

Rosetta Genetics Sample Numbers and Predicted Sequences

Sample Number	Predicted Sequence
miR-2092	UCUCUGGCUAACUAGGGAACCCACUGC
miR-3644	AACUACACACCAGGGCCAGGGGUC
miR-N367	ACUGACCUUUGGAUGGUGCUUCAA
miR-1282	GAACCCACUGCUUAAGCCUCAAUAA
miR-1983	ACCCUCCCAUGCAGAAUAAAACAAAUUA
miR-H1	CCAGGGAGGCGUGCCUGGGC
miR-2875	AGGGGCAAAUGGUACAUCAGGCCA
miR-2111	GUACUGGGUCUCUCUGGUUAGACCAG

Table S5

miRNA sequences predicted from first deep sequencing

miRNA identifier	Sequence
miR-341698	CUGGGAGCUCUCUGGCUA
miR-341699	CUGGGAGCUCUCUGGCUAACUAGGG
miR-341700	UAGACCAGAUCUGAGCCUGGGAGC
miR-341701	GCGUGGCCUGGGUGG
miR-341702	UCACCUAGAACUUUAAAUGC
miR-341703	AAAUGCAUGGGUAAAAGUAGUAGAAGA
miR-341704	CAAUCACCCUCCCAUGCAGA
miR-341707	CACUGACCUUUGGAUGGUGCU
miR-341709	CCAGUUGAGCCAGAUAAGGU

Original, uncropped, and unadjusted images

FIGURE 1A

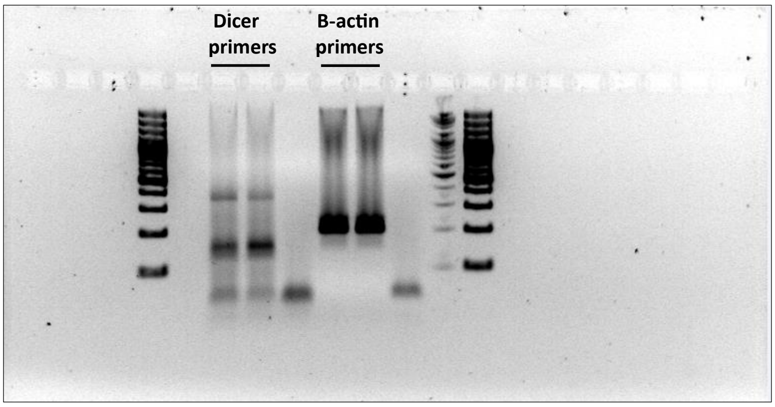

FIGURE 2D

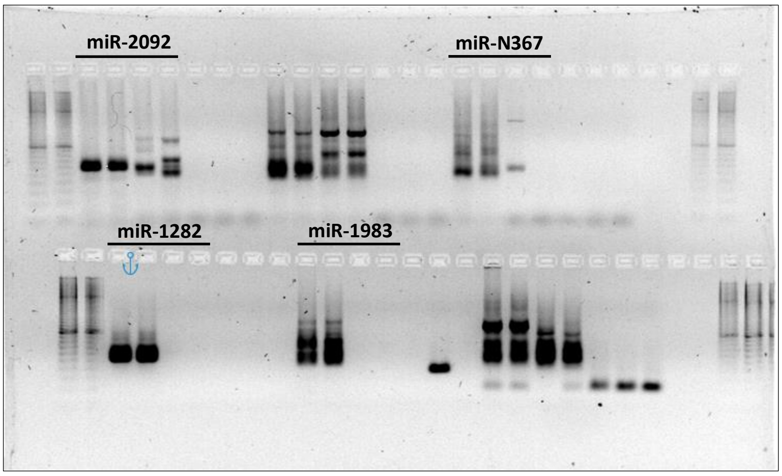

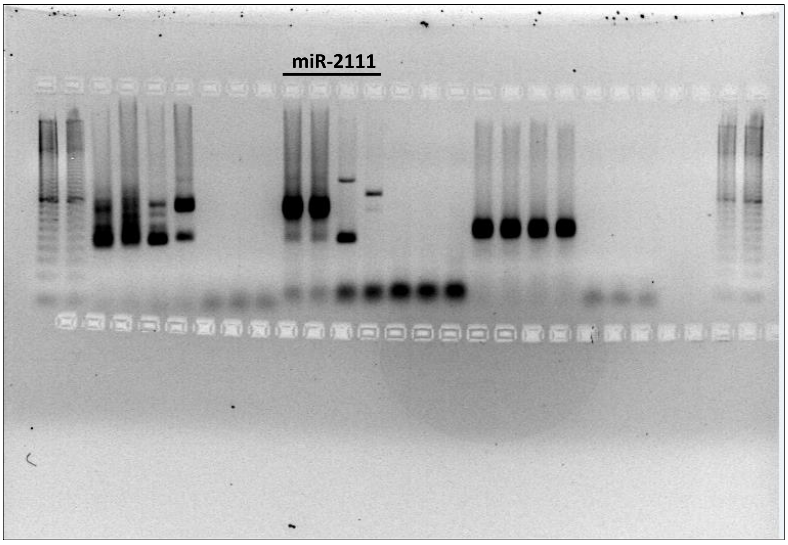

FIGURE 4A

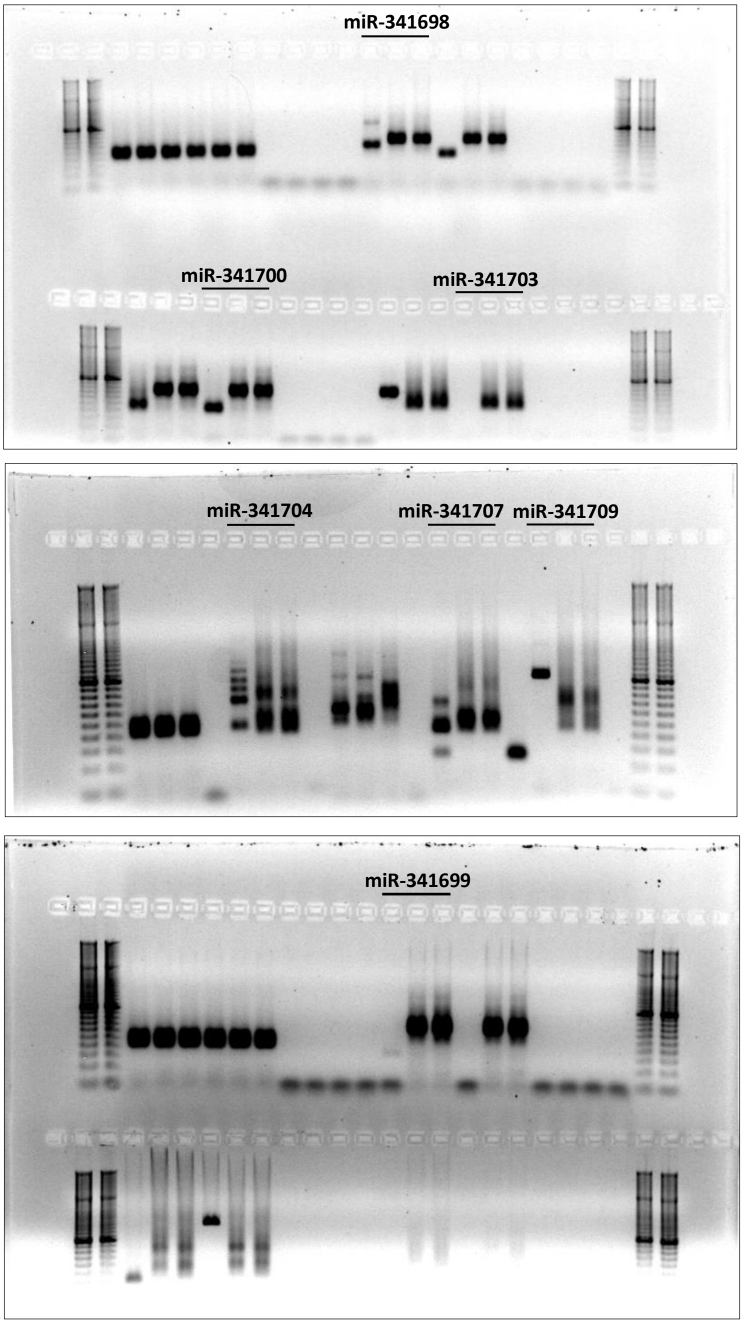

miR-341698
miR-341700
miR-341703
miR-341704
miR-341707
miR-341709
miR-341699

FIGURE 4B

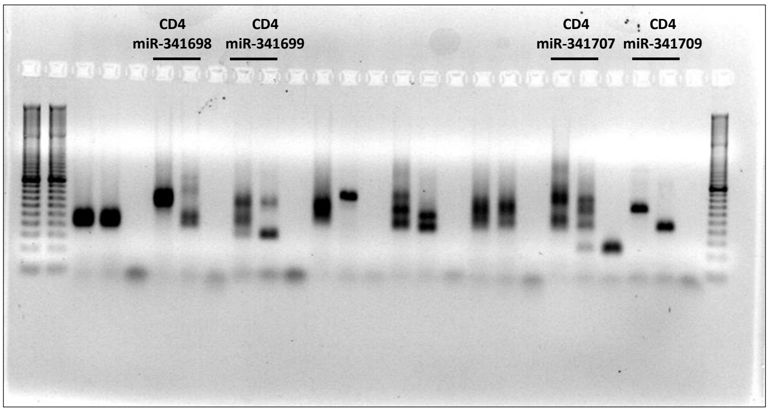
CD4
miR-341698
CD4
miR-341699
CD4
miR-341707
CD4
miR-341709

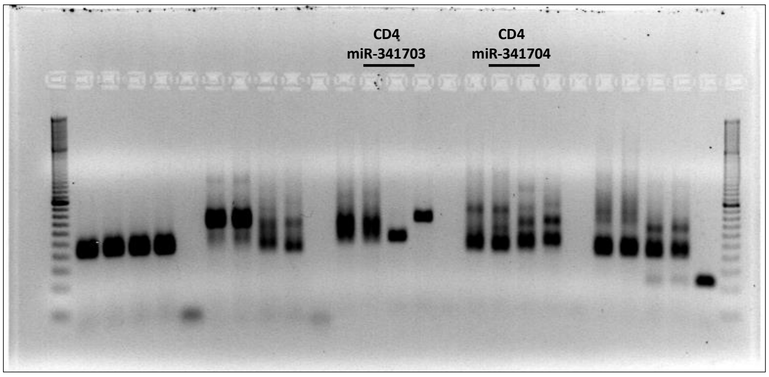
CD4
miR-341703
CD4
miR-341704

FIGURE 5B

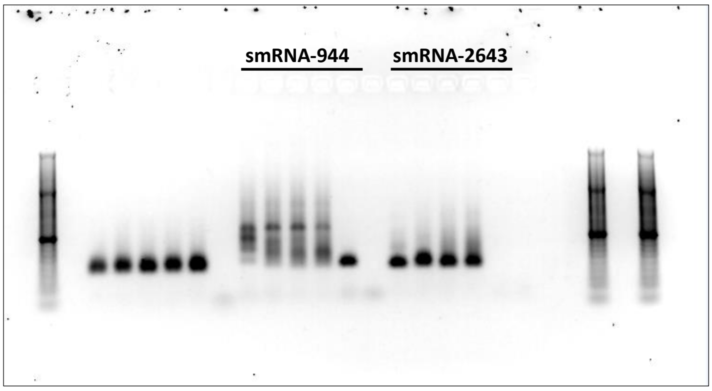
smRNA-944
smRNA-2643

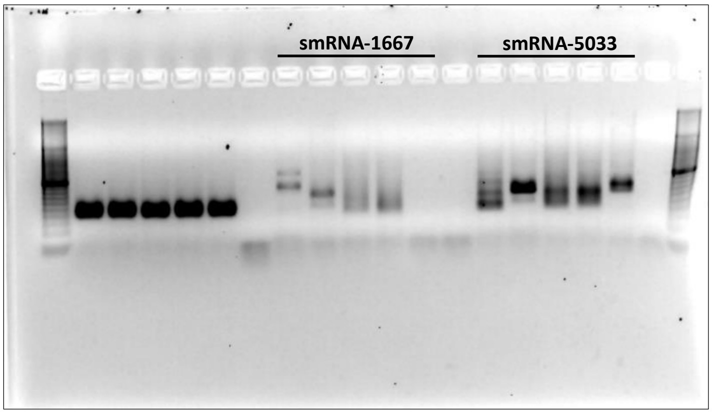
smRNA-1667
smRNA-5033

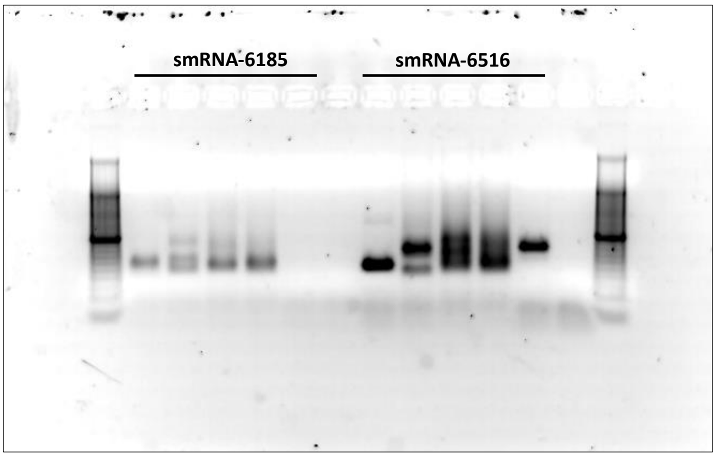
smRNA-6185
smRNA-6516
